# Influenza pneumonia mice under different immune conditions: changes in pulmonary microbiota and metabolites

**DOI:** 10.1128/spectrum.01272-25

**Published:** 2026-01-12

**Authors:** Xindan Liang, Cheng Zhao, Chunjing Chen, Xianggang Zhang, Ling Li, Fangguo Lu

**Affiliations:** 1 School of Integrated Chinese and Western Medicine, Hunan University of Chinese Medicine 118393 , Changsha, Hunan, China; 2 School of Medicine, Hunan University of Chinese Medicine 118393 , Changsha, Hunan, China; Nova Southeastern University, Fort Lauderdale, Florida, USA

**Keywords:** Influenza A virus, influenza pneumonia, immunosuppression, pulmonary microbiota, metabolism

## Abstract

Pneumonia is the most common complication of influenza virus infection and is associated with a high mortality rate in immunocompromised individuals. Currently, neither vaccination nor antiviral therapy has achieved satisfactory therapeutic outcomes. Unlike other organs, the lungs harbor a distinct microbial community due to their unique exposure patterns to airborne particulates and pathogens. However, the characteristic alterations of the pulmonary microbiota and its metabolic products, as well as their potential association with immune suppression in influenza pneumonia, remain inadequately investigated. Mice were rendered immunocompromised through administration of the immunosuppressant cyclophosphamide. Mouse models of influenza pneumonia were established under both immunocompetent and immunosuppressed conditions. Body weight was routinely monitored, and lung histopathology was assessed via hematoxylin and eosin staining. Viral load in lung tissue, serum inflammatory cytokine levels, pulmonary microbiota composition, and lung metabolites were analyzed using RT-qPCR, enzyme-linked immunosorbent assay, 16S rRNA gene sequencing, and untargeted metabolomics, respectively. Spearman correlation analysis was performed to evaluate significant associations between pulmonary microbial taxa and specific metabolites. Influenza virus infection led to a marked reduction in body weight and significantly increased viral load in lung tissue, as well as elevated serum levels of inflammatory cytokines (IL-1β, IL-6, and TNF-α). These effects were more pronounced in immunosuppressed mice, which also exhibited more severe inflammatory and pathological changes in lung tissues. Characteristic shifts in the pulmonary microbiota were observed in the immunosuppressed influenza pneumonia model, particularly involving increased abundance of *Bacteroides* and *Agathobacter*. In addition, key metabolites such as adenosine, adenosine 5′-monophosphate, and xanthine were significantly altered, indicating perturbations in the purine metabolism pathway. Immunosuppressed influenza pneumonia results in more severe inflammatory and pathological lung damage. The observed characteristic changes in the pulmonary microbiota and associated metabolites provide potential microbial and metabolic targets that may contribute to the pathogenesis of severe lung injury in immunocompromised individuals following influenza virus infection.

**IMPORTANCE:**

Taking lung tissue as the entry point, this study directly observes the microbial and metabolite changes in the lungs, distinguishing the effects of different immune states. From this novel perspective, it aims to identify new targets for the treatment of influenza pneumonia.

## INTRODUCTION

Influenza A virus (IAV) infection typically causes symptoms such as fever, rhinorrhea, sore throat, cough, and fatigue. Globally, it is estimated that between 291,000 and 645,000 individuals die each year from influenza-related respiratory complications, with pneumonia being the most common of these complications (1, 2). Elderly individuals, young children, pregnant women, and immunocompromised patients infected with IAV are particularly susceptible to developing pneumonia, which significantly increases the risk of mortality (3). Currently, the primary strategies for prevention and treatment include vaccination and antiviral therapy. However, due to the frequent antigenic drift and shift of circulating viral strains, vaccines must be updated regularly. Although antiviral drugs can reduce the severity and duration of influenza and its complications, they are most effective when administered in the early stages of infection and may lead to the emergence of drug-resistant viral strains. Therefore, there is an urgent need to develop new therapeutic strategies. It is well established that the host immune response plays a critical role in the pathogenesis of influenza (4). Cyclophosphamide, an alkylating agent with myelosuppressive activity, disrupts DNA replication, induces apoptosis, reduces NK cell activity, and suppresses T- and B-cell proliferation, thereby creating an immunosuppressed state. It is widely used to establish murine immunosuppression models, including those for influenza virus infection (5–8). Intraperitoneal administration at defined doses increases murine susceptibility to viral infection, aggravates pathogenicity, and results in more severe pneumonia with extrapulmonary involvement (6–10). In this study, we first established an immunosuppressed mouse model using cyclophosphamide and subsequently intranasal infection with the influenza virus. This model simulates the immunocompromised population infected with IAV, aiming to explore how immune status influences the development and progression of influenza-associated pneumonia (11).

Due to its unique exposure to oxygen and airborne particulates, the lung harbors a microbial community that is markedly distinct from that of other organs (12). External environmental stimuli can alter the composition of the pulmonary microbiota, which in turn influences the development and prognosis of respiratory diseases and can result in alterations of their metabolites (13). In this study, we employed 16S rRNA gene sequencing and untargeted metabolomics to investigate changes in the pulmonary microbiota composition and metabolite profiles in influenza-infected mouse models under different states of immunosuppression. Our aim was to further elucidate the potential mechanisms underlying lung pathology in immunosuppressed hosts with influenza pneumonia, thereby providing theoretical insights and experimental evidence for the identification of novel therapeutic targets.

## MATERIALS AND METHODS

### Animals

A total of 40 BALB/c mice (16–20 g, specific pathogen-free, 20 males and 20 females) were obtained from Hunan Shrek Jingda Experimental Animal (animal production license number SCXK[Xiang] 2019-0004, Animal Batch number 1107271911003527). All mice were housed under standard environmental conditions with a controlled temperature of 24°C–25°C and a 12 h light/dark cycle. During the experiment, the mice had *ad libitum* access to clean drinking water and standard rodent chow.

### Influenza A virus

The mouse-adapted IAV strain (A/PR/8/34) was kindly provided by the Virus Research Laboratory of Hunan Normal University (Changsha, China). The inoculation and passage of the virus strain were conducted in the BSL-2 laboratory at Hunan University of Chinese Medicine, using the 10-day-old chicken embryo allantoic cavity inoculation method (14). The virus suspension with a hemagglutination titer of 1:640 or higher was selected for subsequent experiments. The LD_50_ of the viral stock was calculated as 1 × 10^−3.77^/0.1 mL. The virus was diluted in sterile saline solution to yield 50 LD_50_ per 0.1 mL and kept on ice until use.

### Reagents and chemicals

Cyclophosphamide for injection (BAXTER International Inc, 01407A, America); mouse IL-1β (YJ301814), IL-6 (YJ063159), and TNF-α (YJ002095) enzyme-linked immunosorbent assay (ELISA) kit (Enzyme-linked Biotechnology, Shanghai, China); Total RNA Extraction Kit (TIANGEN, Y1614, Beijing, China); NovoStartSYBR qPCR SuperMix Plus (E096-01A) and NovoScriptPlus All-in-one 1st Strand cDNA Synthesis SuperMix (gDNA purge; EO47-O1B) were purchased from Novoprotein (Suzhou, China).

### Establishment of an animal model

After 3 days of adaptive feeding, the 40 mice were randomly divided into Vehicle/Mock group (*n* = 10), Vehicle/IAV group (*n* = 10), Cyclo/Mock group (*n* = 10), and Cyclo/IAV group (*n* = 10). Following the methodology of our previous study (5), the Cyclo/Mock and Cyclo/IAV groups were administered a single intraperitoneal injection of cyclophosphamide (80 mg/kg), while the Vehicle/Mock and Vehicle/IAV groups received an equal volume of saline. After 24 h, the Vehicle/IAV and Cyclo/IAV groups were intranasally inoculated with 0.05 mL of the prepared influenza virus solution to establish the normal immune and immunosuppressed influenza pneumonia models. On day 5 post-IAV infection, blood samples and lung tissues were collected.

### Body weight measurement and RT-qPCR detection of influenza virus nucleoprotein mRNA expression in lung tissue

Body weight changes in mice from each group were measured and recorded. Four samples were randomly selected from each group to extract total RNA from mouse lung tissue according to the instructions provided by the reagent kit. Subsequently, cDNA was synthesized, using a reverse transcription kit, and amplified using a qPCR kit. β-actin was used as an internal reference, and 2^−∆∆Ct^ was used to analyze the relative expression of mRNA. Primers were synthesized by Tsingke Biotechnology Co., Ltd. (nucleoprotein [NP]: F:CCTGTGTGTATGGACCTGCC R:CTCTTGGGACCACCTTCGTC; β-actin: F:ACATCCGTAAAGACCTCTATGCC R:TACTCCTGCTTGCTGATCCAC). The experiment was performed with three technical replicates.

### Analysis of the hematoxylin and eosin staining of the lungs

Mouse lung tissues were fixed in 4% paraformaldehyde for 1 week. The samples were then dehydrated through a graded ethanol series, embedded in xylene, sectioned, baked, deparaffinized, and stained with hematoxylin and eosin (H&E). Histopathological changes in the lung tissues were observed and documented under a microscope. Lung injury was scored using three sections per group and three randomly selected high-power fields per section (×400). The percentage of damaged alveoli—defined as alveoli containing more than two red blood cells or white blood cells—was calculated as an Index of Quantitative Assessment.

### Enzyme-linked immunosorbent assay

After the mouse serum was directly diluted, the levels of IL-1β, IL-6, and TNF-α in the serum were measured using ELISA kits, following the manufacturer’s instructions. The experiment was performed with three technical replicates.

### 16S rRNA gene sequencing

Four lung tissue samples were selected from each group for DNA extraction using a commercial DNA extraction kit (TianGen, China). The purity and concentration of the extracted DNA were assessed by 1% agarose gel electrophoresis. PCR amplification of the V4 region of the 16S rRNA gene was performed using 10 ng of DNA and specific primers. The primer sequences were: 515F: 5′-GTGCCAGCMGCCGCGGTAA-3′ and 806R: 5′-GGACTACHVGGGTWTCTAAT-3′. The thermal cycling conditions for PCR were as follows: initial denaturation at 98°C for 1 min, followed by 30 cycles of 98°C for 10 s, 50°C for 30 s, 72°C for 30 s, and a final extension at 72°C for 5 min. PCR products were purified using magnetic beads. Sequencing libraries were constructed using the NEBNext Ultra II FS DNA PCR-free Library Prep Kit (New England Biolabs, China). After quality validation by Qubit and qPCR, qualified libraries were sequenced on the NovaSeq 6000 platform using paired-end 250 bp reads. High-throughput sequencing was performed by Novogene Co., Ltd. (Beijing, China).

### Untargeted metabolomics analysis

#### Sample preparation

Lung tissue (100 mg) was ground in liquid nitrogen, and the resulting homogenate was resuspended in 500 μL of pre-chilled 80% methanol. Following vortex mixing and incubation on ice for 5 min, the mixture was centrifuged, and the supernatant was collected. The supernatant was then diluted with mass spectrometry-grade water to adjust the methanol concentration to approximately 53%, followed by a second centrifugation. The final supernatant was analyzed using ultra-performance liquid chromatography-tandem mass spectrometry. High-throughput sequencing was performed by Novogene Co., Ltd. (Beijing, China)

#### Chromatographic and mass spectrometric conditions

Chromatographic conditions: A Vanquish UHPLC system equipped with a Hypersil GOLD column (100 × 2.1 mm, 1.9 μm) was used. The column temperature was maintained at 40 °C. In positive ion mode, 0.1% formic acid (A) and methanol (B) were used as the mobile phases. In negative ion mode, 5 mM ammonium acetate (pH 9.0, A) and methanol (B) were employed. Gradient elution was applied for separation.

Mass spectrometric conditions: Spray voltage: 3.5 kV, sheath gas flow rate: 35 psi, Aux gas flow rate: 10 L/min; capillary temperature: 320°C, S-lens RF level: 60, Aux gas heater temperature: 350°C, and polarity: positive and negative; MS/MS acquisition was performed in data-dependent scan mode.

#### Data preprocessing and analysis

Raw data files (.raw) were imported into Compound Discoverer 3.3 software for peak extraction and quantification based on peak area, followed by integration of target ions. Quality control (QC) samples were used to evaluate data quality. Identified metabolites were annotated using the Kyoto Encyclopedia of Genes and Genomes (KEGG, https://www.genome.jp/kegg/pathway.html), Human Metabolome Database (HMDB), and LIPIDMaps databases (http://www.lipidmaps.org/). Data processing and statistical analyses were performed based on the Linux operating system (CentOS version 6.6) using R (version 3.4.3) and Python (version 3.5.0). Multivariate analyses included partial least squares discriminant analysis (PLS-DA) and orthogonal partial least squares discriminant analysis (OPLS-DA). Differential metabolites were screened based on the criteria of variable importance in projection (VIP) >1 and *P* < 0.05.

### Statistical analysis

Statistical analyses were performed using SPSS version 22.0. Data are presented as mean ± standard deviation ($\bar{x}$ ± s). For normally distributed data, one-way analysis of variance (ANOVA) was used for comparisons among multiple groups. Pairwise comparisons were conducted using either the least significant difference test when homogeneity of variance was assumed, or the Games-Howell test when variance heterogeneity was present. For non-normally distributed data, the Kruskal–Wallis rank-sum test was employed. Differences between groups were considered statistically significant at *P* < 0.05, *P* < 0.01, and *P* < 0.001.

## RESULTS

### Body weight and histopathological changes in lung tissues

**Fig 1:**
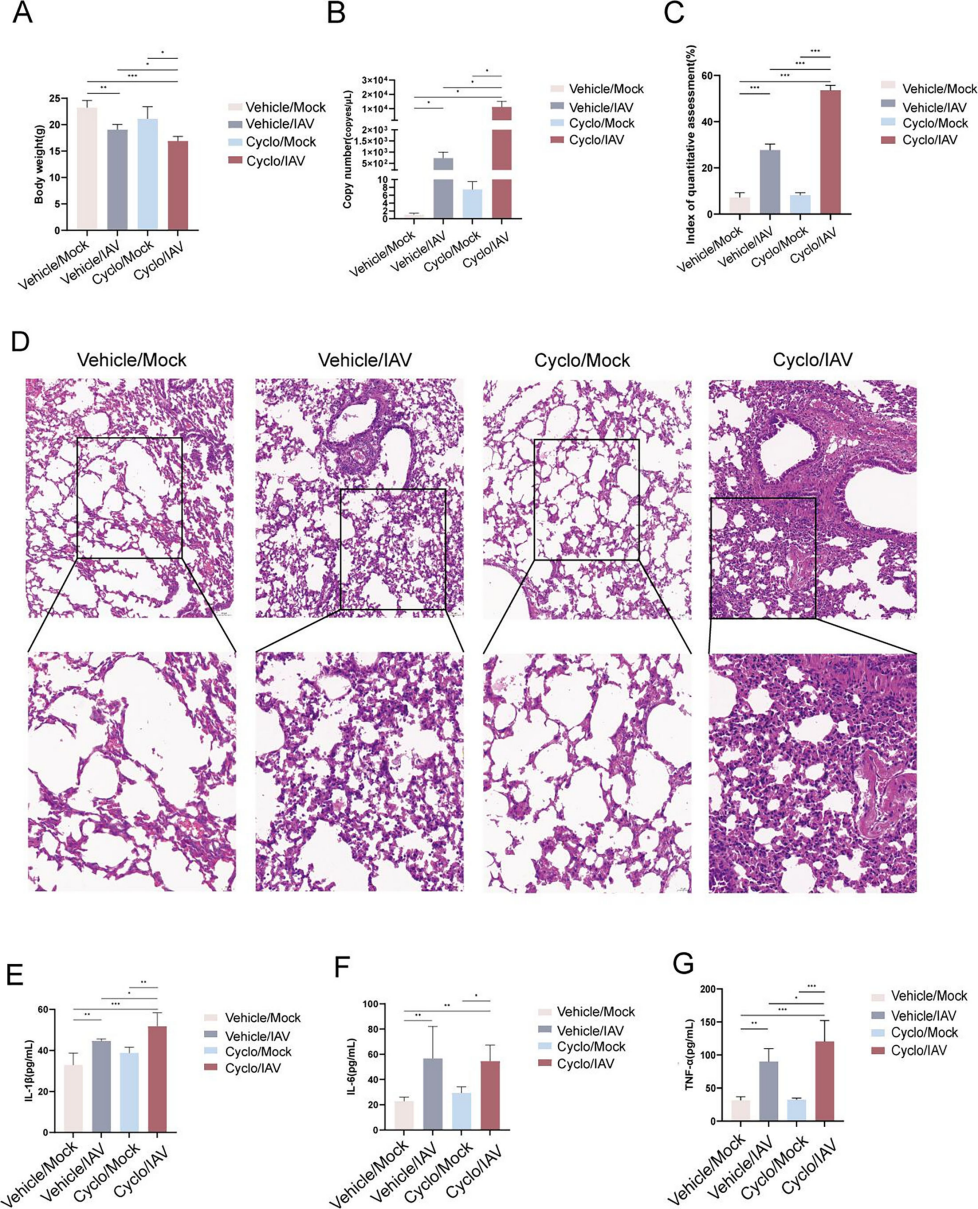
Effects of immune status on influenza-induced pneumonia in mice. (**A**) Changes in body weight among the different groups of mice (mean ± SD, *n* = 6). (**B**) Expression levels of NP in lung tissues across groups (mean ± SD, *n* = 4). (**C and D**) Histopathological changes in lung tissues of each group observed by H&E staining (×200; ×400). (**E–G**) Serum concentrations of cytokines IL-1β, IL-6, and TNF-α in each group determined by ELISA (mean ± SD, *n* = 4).*P < 0.05, **P < 0.01, and ***P < 0.001, as determined by ANOVA followed by Games-Howell post hoc testing. All experiments were performed with three technical replicates.

To investigate the impact of immune status on influenza pneumonia, an immunosuppressive mouse model was established, followed by intranasal infection with the influenza virus. Five days post-infection, compared to the Vehicle/Mock group, mice in the Vehicle/IAV and Cyclo/IAV groups exhibited a significant decrease in body weight (*P* < 0.01 and *P* < 0.001), while body weight remained unchanged in mice treated with cyclophosphamide alone. Moreover, body weight in the Cyclo/IAV group was significantly lower than that in the Vehicle/IAV group (*P* < 0.05; Fig. 1A). On day 5 post-infection, the Cyclo/Mock group showed a higher viral load in lung tissue than the Vehicle/Mock group, but the difference was not statistically significant. Both the Vehicle/IAV and Cyclo/IAV groups demonstrated significantly elevated influenza virus loads in the lungs (*P* < 0.05), with the Cyclo/IAV group showing a higher viral load than the Vehicle/IAV group (*P* < 0.05; Fig. 1B). Histopathological examination of lung tissues was performed using H&E staining. Lung sections from the Vehicle/Mock and Cyclo/Mock groups exhibited intact alveolar architecture with no evident inflammatory cell infiltration. In contrast, lung tissues from the Vehicle/IAV and Cyclo/IAV groups showed marked pathological changes on day 5 post-infection, including alveolar wall thickening, inflammatory cell infiltration, and erythrocyte exudation. These pathological alterations were more severe in the Cyclo/IAV group compared to the Vehicle/IAV group (Fig. 1C and D).

### Expression levels of serum cytokines

The concentrations of serum cytokines IL-1β, IL-6, and TNF-α were quantified using ELISA. Compared with the Vehicle/Mock group, mice in both the Vehicle/IAV and Cyclo/IAV groups showed significantly elevated serum levels of IL-1β, IL-6, and TNF-α at day 5 post-infection (*P* < 0.05, *P* < 0.01, and *P* < 0.001). When compared with the Vehicle/IAV group, the Cyclo/IAV group exhibited significantly higher levels of IL-1β and TNF-α (*P* < 0.05). Furthermore, relative to the Cyclo/Mock group, the Cyclo/IAV group showed a marked increase in IL-1β, IL-6, and TNF-α levels (*P* < 0.05, *P* < 0.01, and *P* < 0.001; Fig. 1E through G). Overall, immunosuppression exacerbates the systemic-inflammatory response in influenza-infected mice, with significantly higher levels of inflammatory factors compared to influenza infection under normal immune conditions.

### Changes in pulmonary microbiota

To investigate the relationship between alterations in the pulmonary microbiota and influenza pneumonia under different immune conditions in mice, we further analyzed the diversity and composition of microbial communities in lung tissues from each group using 16S rRNA sequencing.

#### Diversity analysis

**Fig 2:**
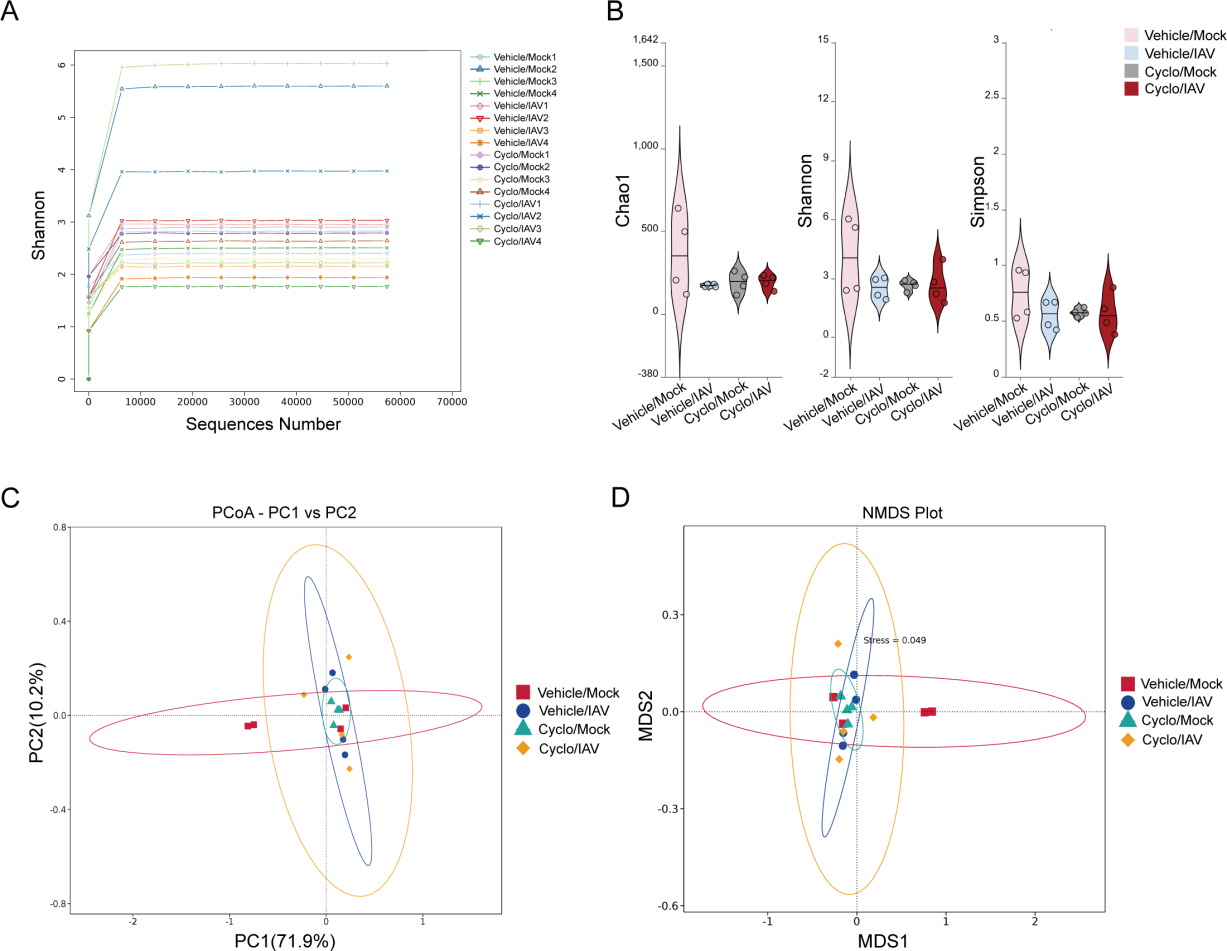
Analysis of α- and β-diversity of lung microbiota across groups. (**A**) Rarefaction curves. (**B**) α-diversity indices. (**C**) PCoA. (**D**) NMDS. A stress value below 0.2 indicates a reliable representation of inter-sample differences.

The rarefaction curves of the Shannon diversity index for the lung microbiota in each group showed a rapid increase in diversity with increasing sequencing depth when the number of sequences was below 6,000. However, as the number of sequences exceeded 6,000, the Shannon index plateaued, indicating that the sequencing depth was sufficient to comprehensively capture the microbial diversity present in the samples, and further analysis was justified (Fig. 2A). The results of α-diversity analysis revealed that the Chao1 richness, as well as the Shannon and Simpson diversity indices, were higher in the Vehicle/Mock group compared to the other three groups. Chao1, Shannon, and Simpson indices suggested that the Vehicle/IAV, Cyclo/Mock, and Cyclo/IAV groups had similarly reduced species richness and evenness, although these differences were not statistically significant (Fig. 2B). β-diversity analysis was performed to compare the microbial community structures among the groups. Principal coordinate analysis (PCoA) and non-metric multidimensional scaling (NMDS) were conducted on the Weighted UniFrac distance, and pairwise comparisons were performed using the Wilcoxon rank-sum test. Significant differences were observed in microbial composition between the Vehicle/IAV and Vehicle/Mock, as well as between the Cyclo/IAV and Cyclo/Mock (*P* < 0.05 and *P* < 0.01; Fig. 2C and D; Table S1). The results showed that viral infection significantly altered the lung microbiota structure in both immunocompetent and immunosuppressed mice. In immunocompetent mice, cyclophosphamide induced marked microbiota shifts.

#### Changes in the relative abundance of the microbiota

**Fig 3:**
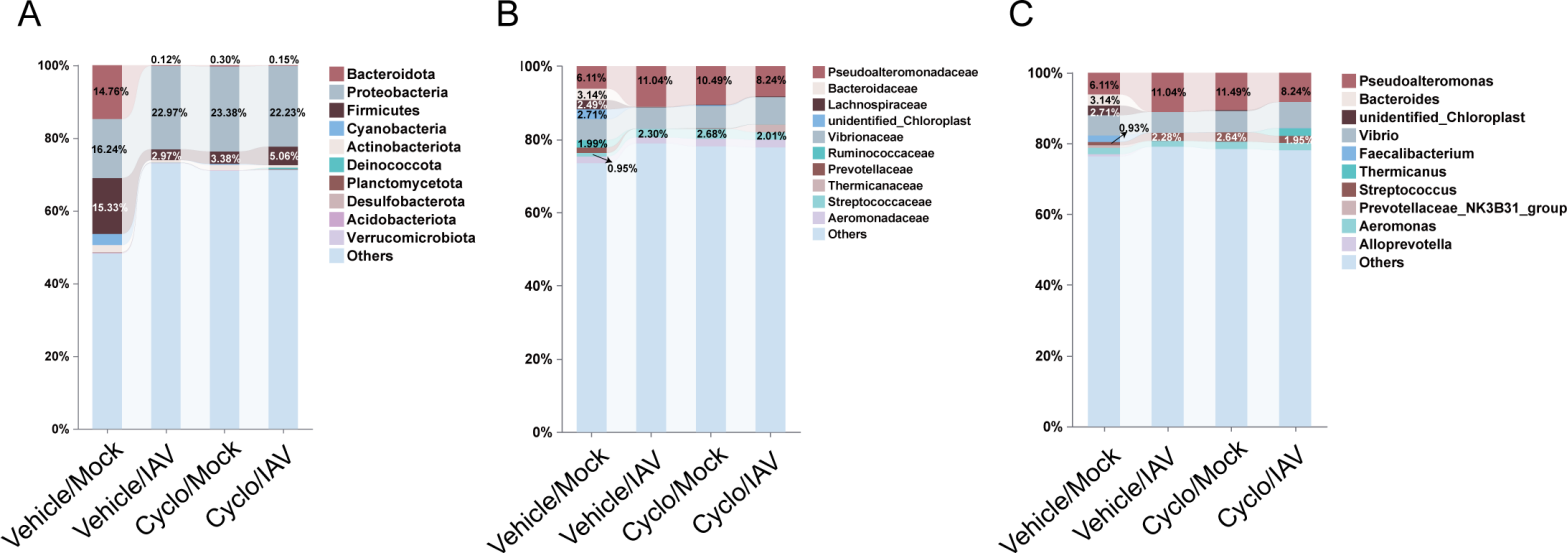
Relative abundance of pulmonary microbiota. (**A**) Phylum. (**B**) Family. (**C**) Genus.

Based on the taxonomic annotation results, the top 10 most abundant taxa at the phylum, family, and genus levels in each group were selected to generate bar plots of relative abundance. At the phylum level, *Proteobacteria*, *Firmicutes*, and *Bacteroidetes* were the most dominant microbial groups in the lung microbiota. Compared with the Vehicle/Mock group, after intervention with influenza virus or cyclophosphamide, the relative abundance of *Firmicutes* and *Bacteroidetes* was decreased, while the relative abundance of *Proteobacteria* was increased in the other groups (Fig. 3A). At the family level, *Bacteroidaceae*, *Pseudoalteromonadaceae*, *Lachnospiraceae*, and *Ruminococcaceae* exhibited relatively high abundance (Fig. 3B). At the genus level, the lung microbiota was primarily composed of *Bacteroides*, *Pseudoalteromonas*, *unidentified_Chloroplast*, and *Faecalibacterium*. Compared with the Vehicle/Mock group, the relative abundances of *Bacteroides* and *unidentified_Chloroplast* were reduced, whereas those of *Pseudoalteromonas* and *Streptococcus* were increased (Fig. 3C).

**Fig 4:**
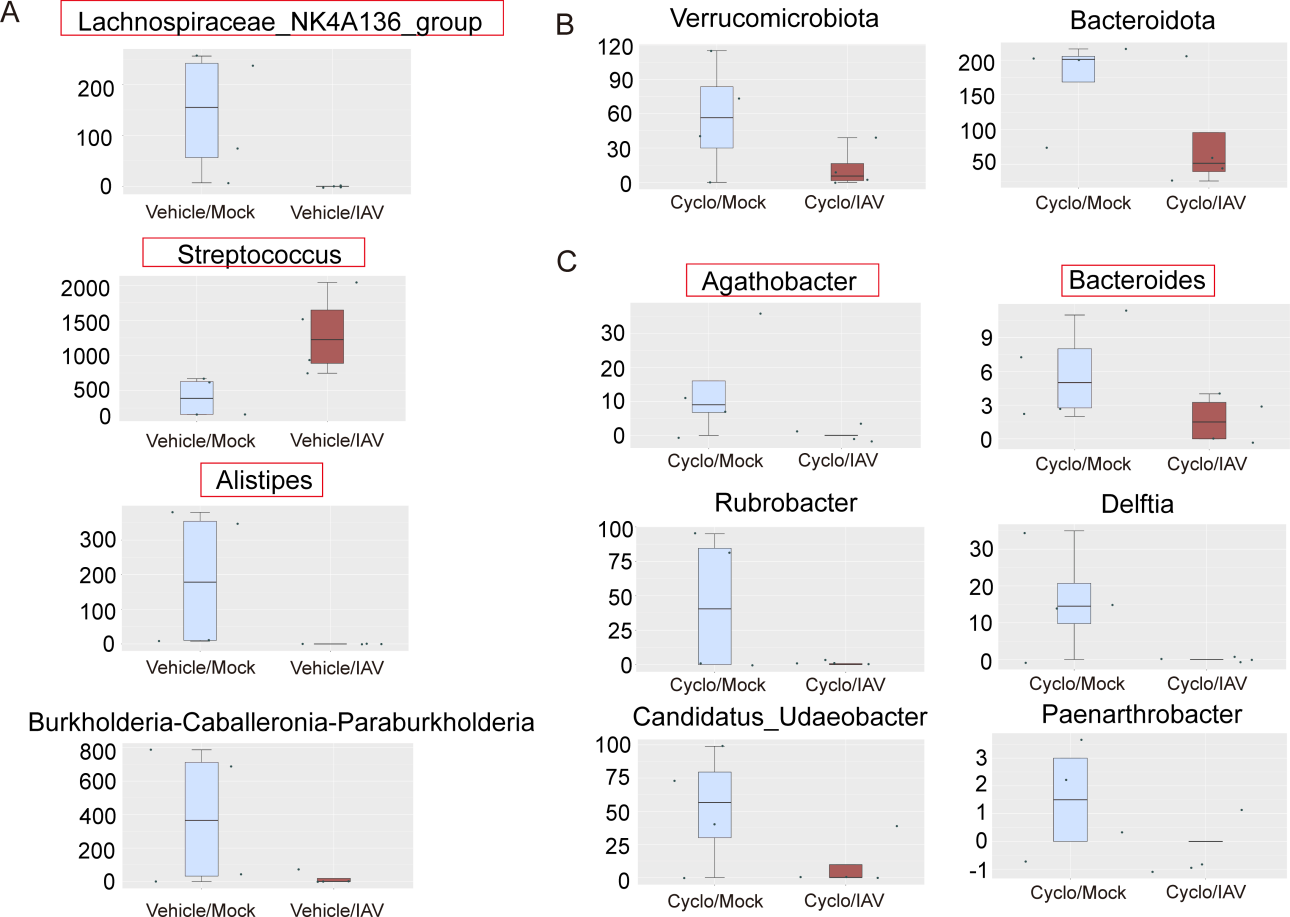
Metastat analysis of differential lung microbiota. (**A**) Genus level: Vehicle/IAV vs Vehicle/Mock. (**B**) Phylum level: Cyclo/IAV vs Cyclo/Mock. (**C**) Genus level: Cyclo/IAV vs Cyclo/Mock.

Metastat analysis: Compared with the Vehicle/Mock group, 29 genera exhibited significant differences in the Vehicle/IAV group. Among them, the relative abundances of *Pseudoalteromonas*, *Streptococcus*, *Marinomonas*, *Ralstonia*, and *Enhydrobacter* were significantly increased, while *Burkholderia-Caballeronia-Paraburkholderia*, *Lysobacter*, *Alistipes*, and *Lachnospiraceae_NK4A136_group* were significantly decreased following IAV infection (Fig. 4A; Table S2). When comparing the Cyclo/IAV group with the Cyclo/Mock group, at the phylum level, the abundances of *Bacteroidota* and *Verrucomicrobiota* were significantly lower in the Cyclo/IAV group (Fig. 4B). At the genus level, eight genera showed significant differences. The relative abundances of *Bacteroides*, *Agathobacter*, *Candidatus_Udaeobacter*, *Rubrobacter*, *Delftia*, and *Paenarthrobacter* were significantly decreased in the Cyclo/IAV group, whereas *Ruminococcus* and *Uruburuella* were significantly increased. Notably, *Bacteroides* was among the top 10 dominant genera in terms of total abundance across all four groups (Fig. 4C; Table S3). These findings indicate that influenza virus infection in immunocompetent mice leads to a significant increase in *Streptococcus* and a marked decrease in *Alistipes* and *Lachnospiraceae_NK4A136_group* in the lungs. In contrast, immunosuppressed mice with influenza pneumonia exhibit significant reductions in microbial genera such as *Bacteroides* and *Agathobacter*.

**Fig 5:**
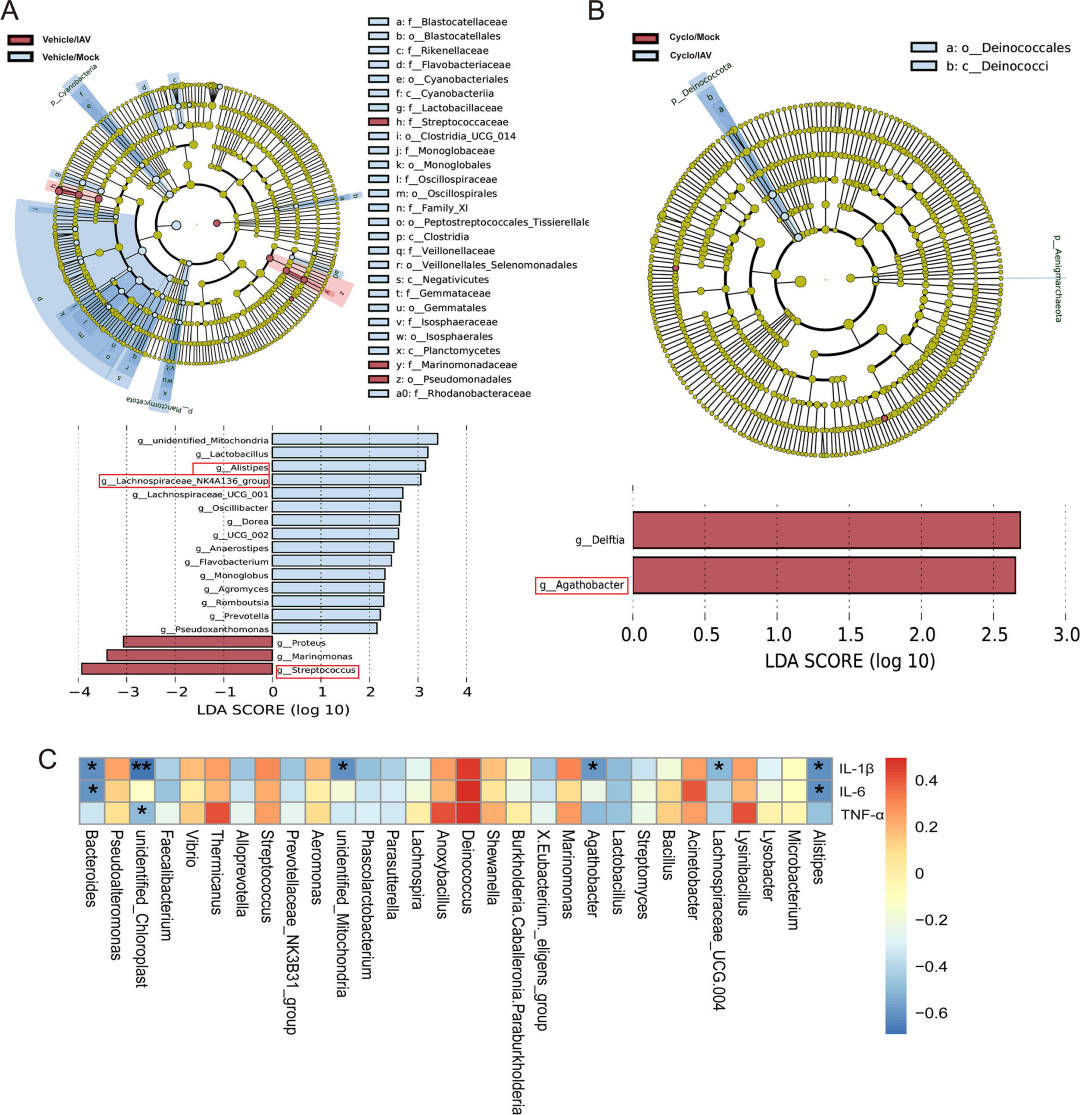
LEfSe analysis of differential lung microbiota and correlation analysis between microbial communities and inflammatory cytokines. (**A**) Vehicle/IAV vs Vehicle/Mock. (**B**) Cyclo/IAV vs Cyclo/Mock. (**C**) Spearman correlation analysis between the top 30 most abundant microbial genera and inflammatory cytokines.*P < 0.05 and **P < 0.01.

Linear discriminant analysis effect size (LEfSe) analysis identified differential taxa between groups as potential biomarkers. At the genus level, a total of 18 significantly different genera were observed between the Vehicle/IAV group and the Vehicle/Mock group (linear discriminant analysis [LDA] score > 2; *P* < 0.05). Several of these, including *Streptococcus*, *Lachnospiraceae_NK4A136_group*, and *Alistipes*, overlapped with the Metastat results. *Streptococcus* had the highest LDA score (3.93) and was identified as a biomarker of the Vehicle/IAV group. *Lachnospiraceae_NK4A136_group* (LDA: 3.05) and *Alistipes* (LDA: 3.15) were biomarkers of the Vehicle/Mock group (Fig. 5A). Between the Cyclo/IAV group and the Cyclo/Mock group, two significantly different taxa were identified—*Agathobacter* (LDA: 2.37) and *Delftia* (LDA: 2.45)—both serving as biomarkers for the Cyclo/Mock group (Fig. 5B).

Spearman correlation analysis was performed between the top 30 genera (based on total abundance across all four groups) and inflammatory cytokines IL-1β, IL-6, and TNF-α. The results revealed a strong correlation between changes in lung microbial composition and abundance and the levels of these inflammatory markers (Fig. 5C). In summary, immunosuppression in influenza virus infection may lead to a significant reduction in bacterial genera such as *Bacteroides*, *Agathobacter*, and *Delftia*, which in turn could result in further elevation of inflammatory cytokines IL-1β, IL-6, and TNF-α, thereby exacerbating lung pathology in the Cyclo/IAV group compared to the Vehicle/IAV group.

### Analysis of differential metabolites in the lung

Data QC was performed using Pearson correlation analysis to ensure the stability and accuracy of the metabolomics results. High Pearson correlation coefficients from both positive and negative electrospray ionization (ESI^+^/ESI⁻) modes in lung tissue samples indicated excellent data quality and reproducibility (Fig. S1).

**Fig 6:**
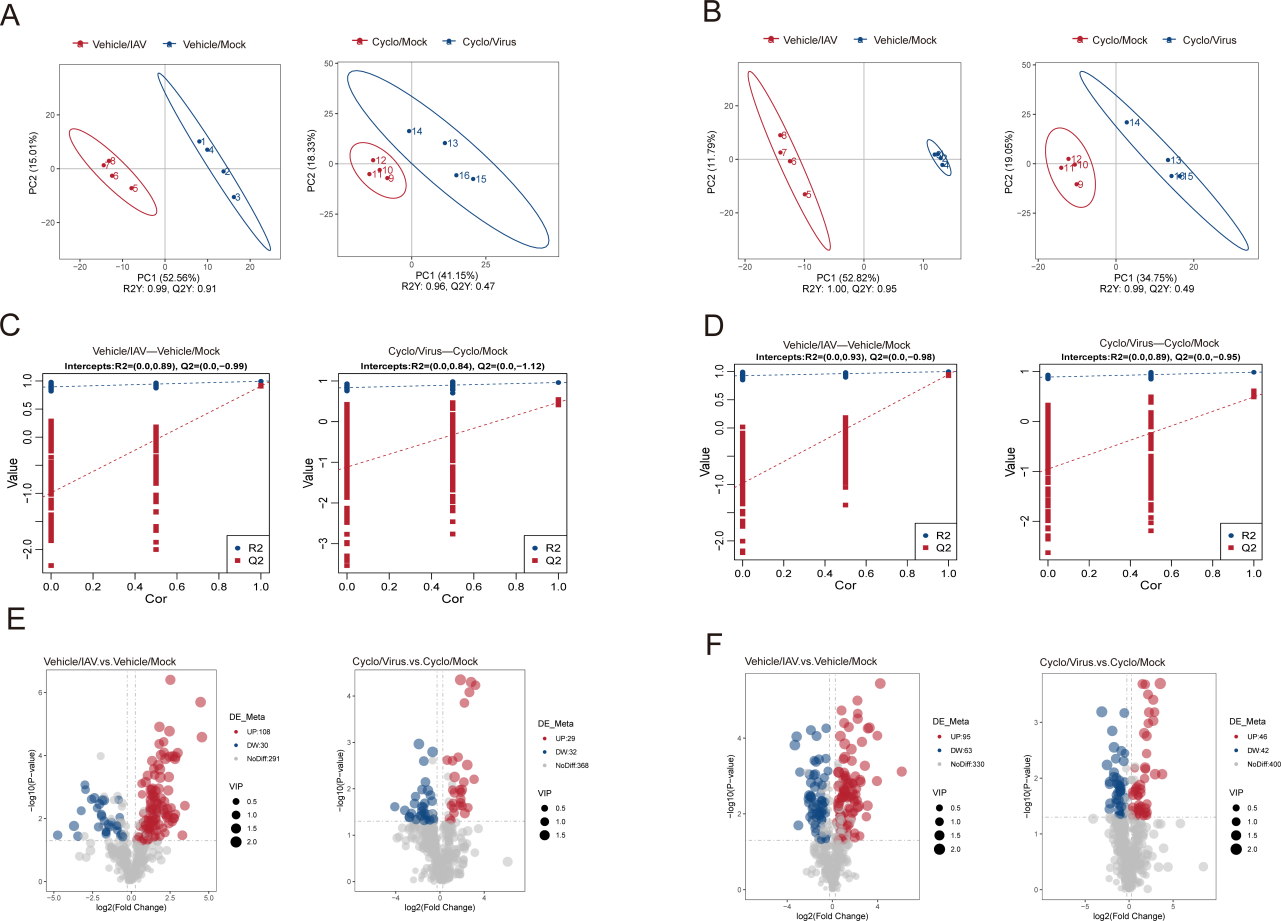
Metabolic model evaluation and differential metabolite screening. (**A and B**) OPLS-DA models of Vehicle/IAV vs Vehicle/Mock and Cyclo/IAV vs Cyclo/Mock. The Vehicle/IAV group and the Cyclo/Mock group are indicated in red, while the Cyclo/IAV group and the Vehicle/Mock group are shown in blue. The X- and Y-axes represent the contribution of each sample to the first two principal components (PC1 and PC2). A: ESI^−^, B: ESI^+^. (**C and D**) Cross-validation plots from 200 permutation tests; C: *R*^2^ = (0.0, 0.89) and *Q*^2^ = (0.0, −0.99); D: *R*^2^ = (0.0, 0.84) and *Q*^2^ = (0.0, −1.12), indicating that the PLS-DA models are not overfitted. C: ESI^−^, D: ESI^+^. (**E and F**) Volcano plots showing pairwise comparisons of lung tissue metabolites between groups. The vertical dashed line indicates the threshold for a twofold change in abundance, and the horizontal dashed line represents the *P*-value cutoff (*P* = 0.05). Student’s *t*-test was used for statistical comparison between groups. Significantly altered metabolites are highlighted in red (upregulated) or blue (downregulated). E: ESI^−^, F: ESI^+^.

Differential metabolites between groups were identified through an OPLS-DA model. In the comparative cohorts, PLS-DA score plots revealed distinct metabolite clustering between the Vehicle/IAV group and the Vehicle/Mock group, as well as between the Cyclo/IAV group and the Cyclo/Mock group. These clustering patterns were consistently observed in both ESI^+^ and ESI⁻ modes (Fig. 6A and B). Subsequently, a metabolomics model was established by fitting the PLS-DA using group labels that were randomly permuted before modeling and prediction. Model performance was evaluated by sevenfold cross-validation, yielding *R*^2^ (goodness of fit) and *Q*^2^ (predictive ability) values. A total of 200 permutation tests were performed to assess model validity. When the *R*^2^ value exceeded the *Q*^2^ value and the intercept of the *Q*^2^ regression line on the Y-axis was less than 0, the model was considered robust and not overfitted. The final PLS-DA model demonstrated satisfactory fitness and excellent predictive performance (Fig. 6C and D). To further investigate these differences, VIP analysis was employed as a key parameter for evaluating differential metabolites. Metabolites meeting the following criteria were considered significantly altered: VIP ≥ 1.0, fold change (FC) ≥ 1.2 or FC < 0.833, and *P* < 0.05. Between the Vehicle/IAV and Vehicle/Mock groups, 296 differential metabolites were identified, including 138 metabolites in ESI^−^ mode (30 downregulated and 108 upregulated) and 158 metabolites in ESI^+^ mode (63 downregulated and 95 upregulated). Between the Cyclo/IAV and Cyclo/Mock groups, 149 differential metabolites were detected (Table S4), comprising 61 metabolites in ESI^−^ mode (32 downregulated and 29 upregulated) and 88 metabolites in ESI^+^ mode (42 downregulated and 46 upregulated; Fig. 6E and F).

**Fig 7:**
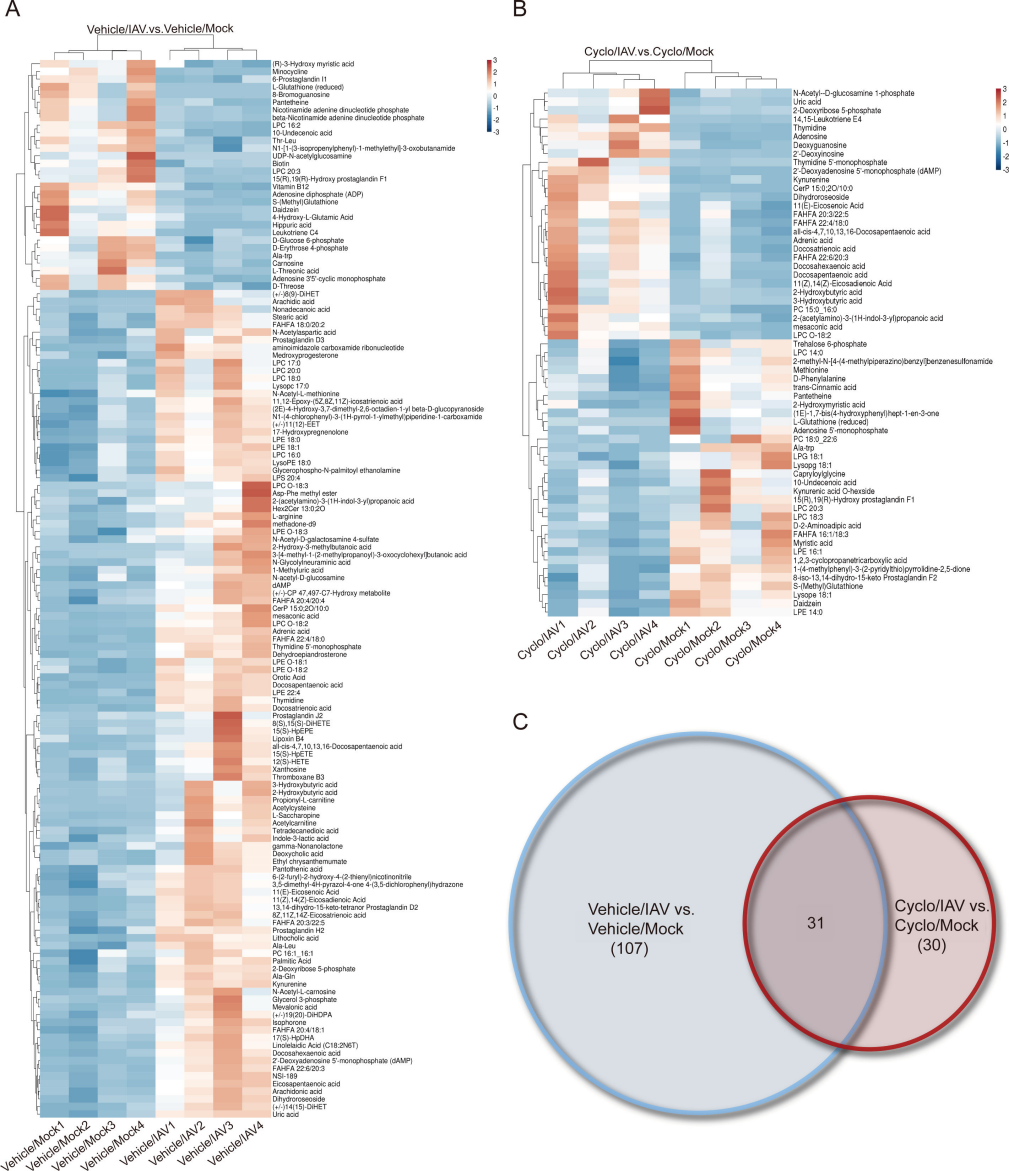
Cluster analysis of differential metabolites and Venn diagram in ESI^−^ mode. (**A and B**) Hierarchical clustering heatmaps of differential metabolites. The vertical axis shows clustering of samples, and the horizontal axis shows clustering of metabolites. Shorter branch lengths indicate higher similarity. Horizontal comparisons reveal the relationships of metabolite abundance patterns across groups. A: Vehicle/IAV vs Vehicle/Mock; B: Cyclo/IAV vs Cyclo/Mock. (**C**) Venn diagram of differential metabolites between Cyclo/IAV vs Cyclo/Mock and Vehicle/IAV vs Vehicle/Mock comparisons.

To clarify the metabolic changes, differential metabolites were independently subjected to clustering analyses in both ESI^−^ (Fig. 7A and B) and ESI^+^ modes (Fig. S2AB). Venn diagrams were performed on 57 specific metabolites unique to the Cyclo/IAV group (Table S5), including 30 in the ESI^−^ mode (Fig. 7C) and 27 in the ESI^+^ mode (Fig. S2C).

**Fig 8:**
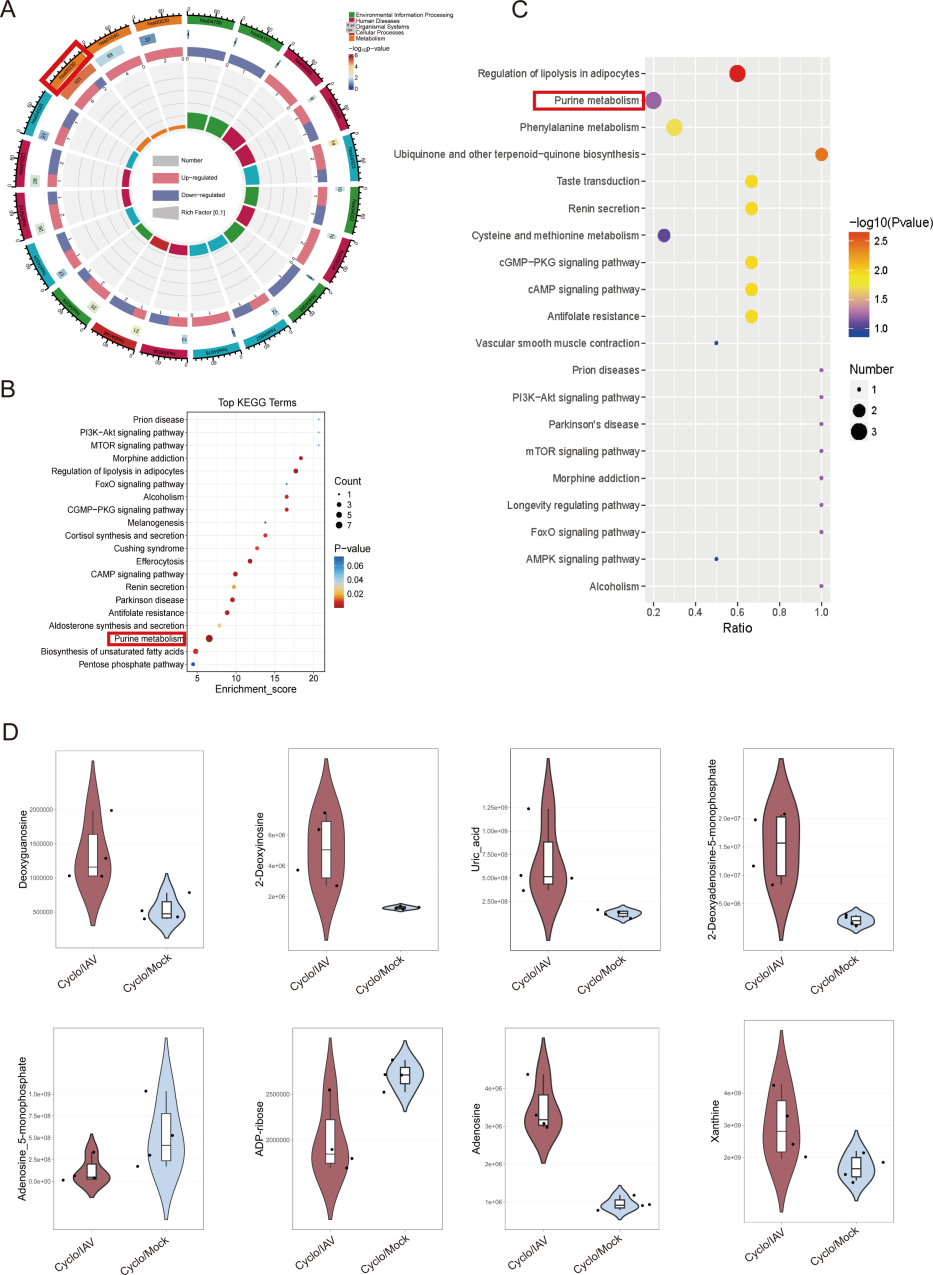
Differential metabolite enrichment analysis. (**A, B, and C**) Enrichment analysis of differential metabolites. A and B: the enrichment results of 149 total metabolites. C: the enrichment analysis of 57 specific metabolites. (**D**) Presentation of differential purine metabolites.

A total of 149 differential metabolites identified in both positive and negative ion modes between the Cyclo/IAV group and the Cyclo/Mock group were subjected to pathway enrichment analysis. The top 20 pathways with the lowest *P*-values were visualized. Among them, purine metabolism (hsa00230, *P* < 0.001, enrichment score: 6.54), biosynthesis of unsaturated fatty acids (hsa01040, *P* < 0.01, enrichment score: 4.79), and pentose phosphate pathway (hsa00030, *P* < 0.05, enrichment score: 4.46) showed significant metabolic alterations, with purine metabolism being the most significantly enriched pathway, involving eight metabolites (Fig. 8A and B). To further investigate characteristic metabolic changes between the Cyclo/IAV group and the Cyclo/Mock group, enrichment analysis was performed on 57 uniquely differential metabolites. The most significantly altered pathways were purine metabolism (*P* < 0.001) and phenylalanine metabolism (*P* < 0.05; Fig. 8C). Notably, adenosine, 2′-deoxyinosine, deoxyguanosine, and adenosine 5′-monophosphate were involved in purine metabolism, with the first three metabolites showing significant upregulation (Fig. 8D).

**Fig 9:**
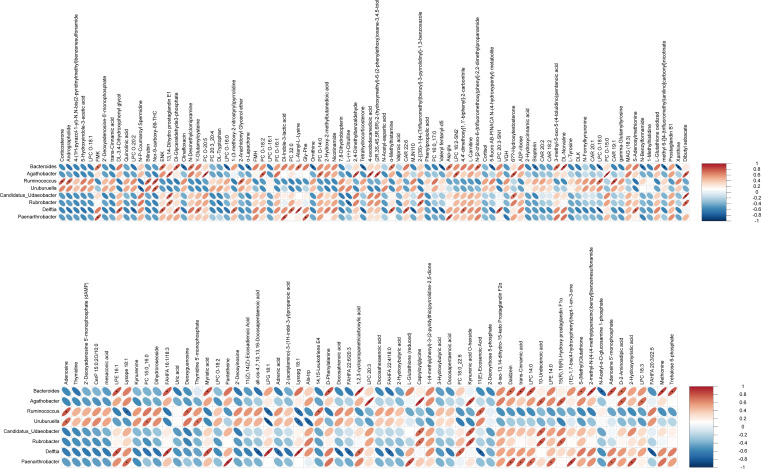
Correlation analysis between differential microbial taxa and metabolites. Heatmaps illustrating correlations between differential microbial taxa and differential metabolites. *P < 0.05.

To further investigate the relationship between pulmonary microbiota and host metabolism, a correlation heatmap was constructed to visualize the interactions between differentially expressed host metabolites and bacterial genera. In this study, among the differential bacterial taxa between the Cyclo/IAV group and the Cyclo/Mock group, *Agathobacter* emerged as a potential biomarker for the Cyclo/Mock group, with a significantly reduced abundance observed in the Cyclo/IAV group. *Agathobacter* abundance was positively correlated with lipid-related metabolites, including PC O-20:5, PC O-18:2, PC O-14:0, PC O-15:0, LPC 20:3, LPC 18:3, as well as 4-oxododecanedioic acid, 13,14-Dihydro prostaglandin E1, D-2-Aminoadipic acid, and (2R,3S,4S,5R,6R)−2-(hydroxymethyl)−6-(2-phenylethoxy)oxane-3,4,5-triol. *Bacteroides* showed the highest overall abundance among all differential metabolites and was significantly negatively correlated with several purine metabolism-related compounds, including adenosine 5′-monophosphate, 2-arachidonyl glycerol ether, α-lapachone, ornithine, tetrahydrocorticosterone, N-acetyl-α-D-glucosamine 1-phosphate, and biopterin. In contrast, *Bacteroides* abundance was positively correlated with purine metabolism intermediates such as xanthine; amino acids and derivatives including methionine, Asp-Glu, DL-norvaline, L-alanyl-L-lysine, α-methylhistamine, and D-2-aminoadipic acid; carbohydrate metabolites such as trehalose 6-phosphate and DL-glyceraldehyde 3-phosphate; phospholipid and fatty acid metabolites such as LPC 14:0, PE 16:1, FAHFA 16:1/18:3, and myristic acid; as well as other compounds like FMK and ENK. Moreover, purine metabolites exhibited significant correlations with the majority of bacterial genera (Fig. 9).

## DISCUSSION

Compared to other body sites, studies of the pulmonary microbiome and metabolome remain in their infancy. The lung employs several selection mechanisms, such as coughing, clearance by alveolar macrophages, mucociliary movement, and the antimicrobial properties of pulmonary surfactant, to maintain a dynamic equilibrium of microbial composition and metabolites. With the ongoing exploration of pulmonary microbial ecology, researchers have found that an imbalance between microbial colonization and clearance in the lungs may be associated with immune function and metabolic processes, thereby contributing to the pathogenesis and progression of pulmonary diseases (15). Our previous work (16) demonstrated that influenza virus infection disrupts gut microbial homeostasis, characterized by an increased relative abundance of *Proteobacteria*, a reduced *Firmicutes/Bacteroidetes* ratio, and marked depletion of beneficial genera such as *Coprococcus*, *Lactobacillus*, and *Prevotella*. Moreover, gut microbial alterations were significantly associated with the expression of the chemokines CCL5 and CXCL10, suggesting that influenza virus may modulate chemokine production through microbiota-mediated mechanisms, thereby contributing to immune–inflammatory injury. Research on the lung microbiota and its metabolites provides a more direct perspective on influenza pneumonia, yet their interactions with host immune status and their mechanistic roles in disease progression remain to be fully elucidated. Therefore, this study established models of influenza pneumonia under normal and immunosuppressed conditions to investigate the alterations in pulmonary microbiota and metabolites following influenza virus infection under different immune statuses, aiming to further elucidate the role of immune state in influenza pneumonia.

The pathogenesis and clinical prognosis of influenza pneumonia are influenced by the host’s immune response. Extensive research has been dedicated to understanding how various components of the host’s innate and adaptive immune responses contribute to the response to influenza virus infection (17, 18). Various pattern recognition receptors, such as Toll-like receptors, retinoic acid-inducible gene I, and NOD-like receptors, recognize the influenza virus as a foreign entity, leading to the production of pro-inflammatory cytokines and type I interferons. These responses form the first line of defense against influenza infection and trigger the adaptive immune response, including virus-specific B cell and T cell responses (19). Cyclophosphamide-induced immune dysfunction preferentially depletes proliferating lymphocytes, particularly CD4^+^/CD8^+^ T cells and regulatory T cells (Tregs). Depletion of CD4^+^/CD8^+^ T cells impairs immune regulation, delays viral clearance, and sustains antigenic stimulation, thereby promoting excessive activation of monocytes and macrophages. Under physiological conditions, Tregs restrain innate immune hyperactivation. Their depletion removes this regulatory brake and permits uncontrolled production of innate cytokines such as IL-1β, TNF-α, and IL-6 (20, 21). In parallel, cyclophosphamide disrupts the gut microbiota, reducing beneficial taxa and altering host metabolic homeostasis. These microbiota-derived perturbations can further amplify systemic-inflammatory responses in the lung via the gut-lung axis (22, 23). Cyclophosphamide has been employed to establish an immunosuppressed IAV-infected murine pneumonia model, where it aggravates pulmonary pathology and causes extrapulmonary organ damage. On this basis, a cyclophosphamide-induced influenza-associated encephalitis model was developed to investigate the therapeutic mechanisms of Chinese herbal compounds (11, 24, 25). Microbiome analysis of pulmonary samples from influenza-infected mice identified distinct microbial community shifts after infection (26). The present study centers on immunocompromised populations with increased susceptibility, examining concurrent alterations in pulmonary microbiota and metabolic profiles. The lung microbiota primarily interacts with lung epithelial cells and alveolar macrophages, and its symbiotic state may participate in immune responses, prevent inflammation, and maintain a clean and safe lung environment (27). Individuals lacking lung-specific microbiota and/or their metabolites exhibit mucosal immune characteristics with a Th17/neutrophil phenotype, while suppressing innate immune function, suggesting a potential immune regulatory and airway inflammation regulation mechanism (28). Dickson et al. demonstrated in an *in vivo* experiment that the structure and diversity of the lung microbiota were significantly correlated with inflammatory cytokine levels in mice (29). Short-chain fatty acids (SCFAs) produced by the lung microbiota, such as acetate, propionate, and butyrate, can suppress the expression of NLRP3, Caspase-1, IL-1β, and IL-18, thereby reducing the levels of inflammatory cytokines (30). These findings reveal that different immune states and the prognosis of influenza pneumonia are closely linked to the levels of lung microbiota and their associated metabolites.

Based on previous studies and the literature (5, 7, 11, 31–34), pulmonary viral loads following PR8 infection peak earlier than the onset of maximal histopathological injury. Under cyclophosphamide-induced immunosuppression, significant inflammatory cell infiltration and differences in pathology scores can be consistently observed by day 5 post-influenza infection. This time point captures the acute inflammatory phase and immune activation while minimizing confounding influences from later-stage events such as secondary bacterial infection, extensive tissue damage, or mortality-related bias.

This study demonstrated that, compared to the Vehicle/Mock group, the Vehicle/IAV group exhibited significantly elevated expression levels of the inflammatory cytokines IL-1β, IL-6, and TNF-α. Similarly, the Cyclo/IAV group showed significantly higher levels of these cytokines compared to the Cyclo/Mock group. Furthermore, relative to the Vehicle/IAV group, the Cyclo/IAV group exhibited markedly increased expression of IL-1β and TNF-α. Associated with viral load and histopathological observations from HE staining, it is still insufficient to meet the diagnostic criteria for “cytokine storm syndrome,” so we will more cautiously describe it as “systemic-inflammatory response.” The above results indicate that influenza virus infection under immunosuppression causes a stronger systemic inflammatory response, and the lungs of mice exhibit more severe immunopathological damage.

Moreover, influenza virus infection led to significant alterations in lung microbiota composition in mice with different immune statuses. Further investigation indicated that, in immunosuppressed mice, the observed exacerbated pathological damage may be attributed to unique microbiota structural changes and associated metabolic alterations, beyond those induced by viral infection alone.

This study analyzed the lung microbiota structure and found that the abundance of *Bacteroides* and *Agathobacter* significantly decreased in immunosuppressed mice infected with the influenza virus, and these decreases were significantly negatively correlated with inflammatory cytokines. *Bacteroides* is one of the most abundant gram-negative anaerobic bacteria in the human gut microbiome, and it can also be present in the lungs. Tarabichi Y et al. confirmed through nasal 16S rRNA sequencing that in healthy individuals aged 18-65, the relative abundance of *Bacteroides* significantly increased following intranasal inoculation of live attenuated influenza vaccine, affecting the host immune system (35). *Bacteroides* ovatus has shown significant correlations with IgA antibody responses to H1N1 and H3N2 influenza viruses in both univariate and multivariate analyses, likely due to the complementary immune response mechanisms between probiotic strains and proteins (36). Influenza infection can lead to a reduction in the abundance of *Bacteroides* in the gut. Administration of *Bacteroides dorei* reduced the production of cytokines (IL-1β, IL-6, TNF-α, etc.) in the lungs and serum induced by influenza infection, which may alleviate excessive inflammatory responses and improve immune pathological damage (37). *Agathobacter*, a member of the Firmicutes phylum, is a common anaerobic bacterium in the human gut. It has been mainly studied as a beneficial bacterium in the gut microbiota, and its abundance is reduced in neurological diseases such as Alzheimer’s disease (38), spinal cord injury (39), and lacunar infarction (40), as well as in hepatitis B and liver cancer (41). Research on pulmonary microbiota in influenza infection is limited and requires further investigation. Both *Bacteroides* and *Agathobacter* produce SCFAs, which play important roles in maintaining gut homeostasis and immune function (42, 43). The relative abundance of *Bacteroides* and *Agathobacter* in the lung tissues of mice treated with cyclophosphamide combined with influenza virus infection was significantly lower than that in the cyclophosphamide group, with a significant increase in inflammatory cytokines. This finding suggests that the decrease in the abundance of *Bacteroides* and *Agathobacter* may promote the excessive release of inflammatory cytokines in immunocompromised populations, leading to more severe immune pathological damage compared to individuals with normal immune function.

Metabolomics analysis constructed a metabolic pathway network using KEGG. The differentially expressed metabolites in the Cyclo/IAV group and the cyclophosphamide group were primarily enriched in purine metabolism, biosynthesis of unsaturated fatty acids, and pentose phosphate pathway, consistent with changes in metabolic pathways annotated in other studies (44, 45). Among them, 57 specific metabolites with altered levels were mainly enriched in purine metabolism, so the focus was placed on this pathway. Of the eight differentially expressed purine metabolites annotated, six showed a significant increase. A study conducted untargeted metabolomics sequencing on serum from patients infected with H3N2 and found persistent changes in the purine metabolism pathway, with metabolites such as inosine, hypoxanthine, and xanthine remaining significantly elevated 21–27 days post-fever (46). Similarly, *in vitro* studies showed that primary human airway epithelial cells infected with IAV could induce the synthesis of purine-related metabolites (47). After H1N1 (PR8) virus infection in mice, differential purine and pyrimidine metabolites were identified in both the lung and bronchoalveolar lavage fluid, and an increase in purine nucleotides was observed in epithelial and immune cells in the airways during inflammation. The study highlighted that adenosine monophosphate is a candidate biomarker for airway inflammation, showing a decreasing trend unlike most other purine and pyrimidine metabolites, with cAMP also showing a downward change. In this study, adenosine 5′-monophosphate displayed a similar decreasing trend (45). This suggests that purine metabolism is significantly associated with influenza pneumonia in an immunosuppressed state, and modulating purine metabolic pathways may improve the severe pulmonary pathological damage. The association between microbiomics and metabolomics results revealed key core genera identified in the lungs of immunosuppressed influenza pneumonia mice, including *Bacteroides*, *Agathobacter*, *Uruburuella*, *Candidatus_Udaeobacter*, *Rubrobacter*, *Delftia*, and *Paenarthrobacter*. These genera were significantly correlated with fatty acid metabolites, tryptophan metabolites, tyrosine metabolites, glutathione metabolites, steroid hormone metabolites, and were also significantly correlated with differentially expressed purine metabolites (adenosine, adenosine 5′-monophosphate, and xanthine). Hosts with different phenotypes carry distinct microbiome abundances and structures, which, in turn, influence their own and the host’s metabolism. This study suggests that specific purine metabolites may serve as biomarkers to characterize the altered metabolic phenotype in immunosuppressed influenza pneumonia, impacting the host’s physiological functions through a series of signaling effects.

In summary, this study, through 16S rRNA sequencing and metabolomic analyses, revealed characteristic alterations in pulmonary microbiota and their associated metabolites in an immunosuppressed mouse model of influenza pneumonia. These findings provide microbiota- and metabolite-related targets potentially involved in the severe lung pathology observed in immunocompromised individuals following influenza virus infection.

Looking ahead, we acknowledge that the cyclophosphamide-induced immunosuppressed mouse model represents an artificial approach to mimic compromised host immunity and has inherent limitations. Although it is widely used to investigate susceptibility to severe influenza infection and for antiviral drug evaluation, it does not fully recapitulate the multifactorial immunosenescence seen in older individuals. Future studies will incorporate aged mouse models to further validate our observations and enhance relevance to human physiology. Further research is needed to elucidate the specific regulatory mechanisms by which influenza pneumonia under immunosuppressive conditions affects the gut microbiota. This includes both the direct and indirect impacts on specific bacterial communities, as well as the interactions between these microbial shifts and the host immune responses and metabolic state. Future studies employing microbiota transplantation as an adjunct approach are expected to uncover the mechanistic roles of microbial alterations in influenza pneumonia and offer new insights for clinical intervention.

## SUPPLEMENTAL MATERIAL

Figure S1 spectrum.01272-25-s0001.tif Evaluation of the metabolic model in positive ion mode and clustering of differential metabolites.

Figure S2 spectrum.01272-25-s0002.tif Cluster Analysis of Differential Metabolites and Venn Diagram in ESI^+^ mode.

Supplemental legends spectrum.01272-25-s0003.docx Legends for Tables S1 to S5, and Figures S1 and S2.

Table S1 spectrum.01272-25-s0004.xlsx Beta diversity analysis between groups.

Table S2 spectrum.01272-25-s0005.xlsx Metastat analysis of differential microbial taxa between the Vehicle/IAV group and the Vehicle/Mock group.

Table S3 spectrum.01272-25-s0006.xlsx Metastat analysis of differential microbial taxa between the Cyclo/IAV group and the Cyclo/Mock group.

Table S4 spectrum.01272-25-s0007.xlsx Differential metabolites between the Cyclo/IAV group and the Cyclo/Mock group.

TABLE S5 spectrum.01272-25-s0008.xlsx Differential metabolites specifically altered between the Cyclo/IAV group and the Cyclo/Mock group.

ASM does not own the copyrights to Supplemental Material that may be linked to, or accessed through, an article. The authors have granted ASM a non-exclusive, world-wide license to publish the Supplemental Material files. Please contact the corresponding author directly for reuse.
