# Supplementary figures and images for: Influenza pneumonia mice under different immune conditions: changes in pulmonary microbiota and metabolites
Source: Microbiol Spectr. 2026 Jan 12;14(2):e01272-25. doi: 10.1128/spectrum.01272-25 (PMC12889112; doi:10.1128/spectrum.01272-25)

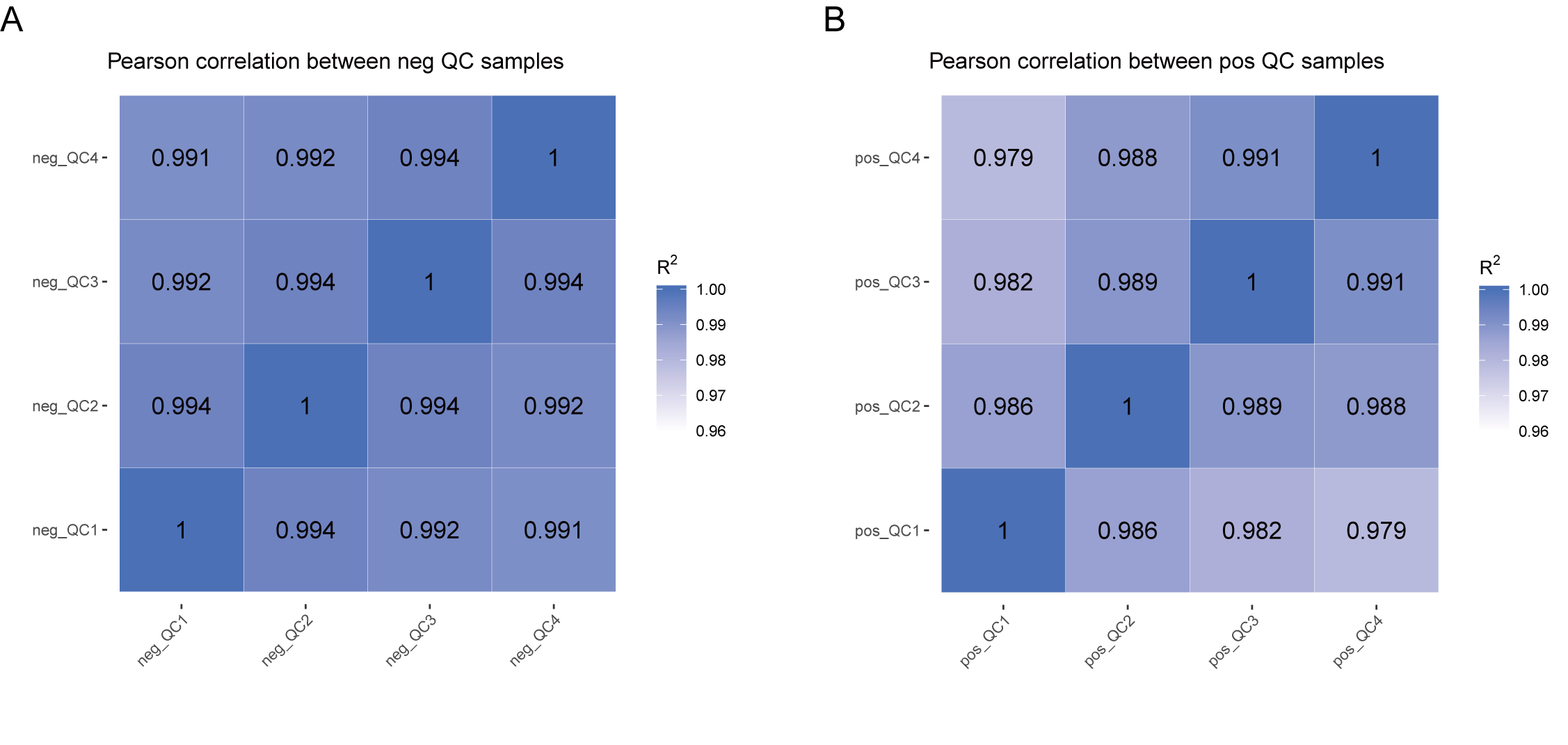

Supplement: Figure S1 — Evaluation of the metabolic model in positive ion mode and clustering of differential metabolites. [file spectrum.01272-25-s0001.tif]

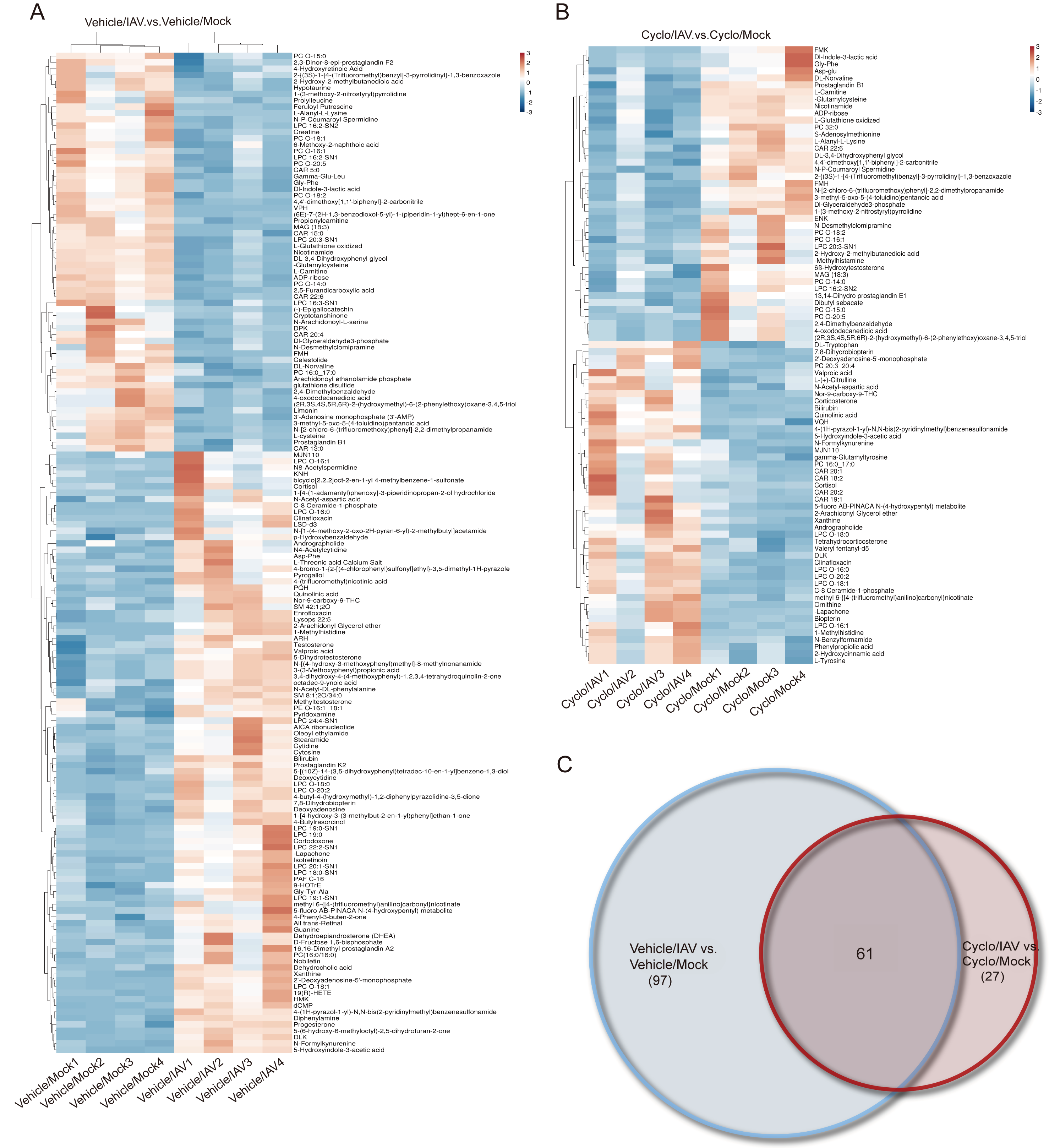

Supplement: Figure S2 — Cluster Analysis of Differential Metabolites and Venn Diagram in ESI+ mode. [file spectrum.01272-25-s0002.tif]
