# Supplementary material for: Influenza pneumonia mice under different immune conditions: changes in pulmonary microbiota and metabolites
Source: Microbiol Spectr. 2026 Jan 12;14(2):e01272-25. doi: 10.1128/spectrum.01272-25 (PMC12889112; doi:10.1128/spectrum.01272-25)
Supplement: Supplemental legends — Legends for Tables S1 to S5, and Figures S1 and S2. [file spectrum.01272-25-s0003.docx]

Supplementary Material

# Supplementary Figures and Tables

## Supplementary Figures

**Supplementary Figure 1.** Quality control of data assessed by Pearson correlation analysis.

(A) ESI-; (B) ESI+

**Supplementary Figure 2.** Cluster Analysis of Differential Metabolites and Venn Diagramin in ESI+ mode.

(A, B) Hierarchical clustering heatmaps of differential metabolites. The vertical axis shows clustering of samples, and the horizontal axis shows clustering of metabolites. Shorter branch lengths indicate higher similarity. Horizontal comparisons reveal the relationships of metabolite abundance patterns across groups. A: Vehicle/IAV vs. Vehicle/Mock;B: Cyclo/IAV vs. Cyclo/Mock.(C) Venn diagram of differential metabolites between Cyclo/IAV vs. Cyclo/Mock and Vehicle/IAV vs. Vehicle/Mock comparisons.

## Supplementary Tables

**Supplementary Table 1.** Beta diversity analysis between groups

ns P > 0.05, *P < 0.05, **P < 0.01.

**Supplementary Table 2.** Metastat analysis of differential microbial taxa between the Vehicle/IAV group and the Vehicle/Mock group

**Supplementary Table 3.** Metastat analysis of differential microbial taxa between the Cyclo/IAV group and the Cyclo/Mock group

**Supplementary Table 4.** Differential metabolites between the Cyclo/IAV group and the Cyclo/Mock group

**Supplementary Table 5.** Differential metabolites specifically altered between the Cyclo/IAV group and the Cyclo/Mock group
